# Knockdown of MCM8 functions as a strategy to inhibit the development and progression of osteosarcoma through regulating CTGF

**DOI:** 10.1038/s41419-021-03621-y

**Published:** 2021-04-07

**Authors:** Zhinan Ren, Jun Li, Shanwen Zhao, Qi Qiao, Runguang Li

**Affiliations:** 1 Department of Orthopedics, The First Affiliated Hospital of Zhengzhou University, Zhengzhou, 450052 China; 2 Department of Orthopedics, The Second Affiliated Hospital of Anhui Medical University, 678 Furong, Hefei, 230601 China; 3 Department of Foot and Ankle Surgery, Center for Orthopaedic Surgery, The Third Affiliated Hospital of Southern Medical University, Guangzhou, 510610 China; 4 Orthopaedic Hospital of Guangdong Province, Guangzhou, 510630 China; 5 Academy of Orthopaedics, Guangdong Province, Guangzhou, 510630 China; 6 Guangdong Provincial Key Laboratory of Bone and Joint Degenerative Diseases, Guangzhou, 510515 China; 7 Department of Orthopedics, Linzhi People’s Hospital, Linzhi, 860000 China

**Keywords:** Oncogenes, Sarcoma

## Abstract

Osteosarcoma is the most common primary malignant tumor of bone derived from osteoblasts, which is a noteworthy threat to the health of children and adolescents. In this study, we found that MCM8 has significantly higher expression level in osteosarcoma tissues in comparison with normal tissues, which was also correlated with more advanced tumor grade and pathological stage. In agreement with the role of MCM proteins as indicators of cell proliferation, knockdown/overexpression of MCM8 inhibited/promoted osteosarcoma cell proliferation in vitro and tumor growth in vivo. Also, MCM8 knockdown/overexpression was also significantly associated with the promotion/inhibition of cell apoptosis and suppression/promotion of cell migration. More importantly, mechanistic study identified CTGF as a potential downstream target of MCM8, silencing of which could enhance the regulatory effects of MCM8 knockdown and alleviate the effects of MCM8 overexpression on osteosarcoma development. In summary, MCM8/CTGF axis was revealed as critical participant in the development and progression of osteosarcoma and MCM8 may be a promising therapeutic target for osteosarcoma treatment.

## Introduction

Osteosarcoma is the most common primary malignant tumor of bone derived from osteoblasts, the main predisposing population of which is children and adolescents^1,2^. Osteosarcoma mostly occurs in the epiphysis of the long diaphysis, which grows rapidly and characterized by strong invasiveness. Therefore, lung metastases may appear in the early stage of the disease^3^. In recent years, with the development of limb salvage surgery, chemotherapy, and radiation therapy, the overall survival rate of osteosarcoma patients has reached 60–75%^4^. However, the survival rate of osteosarcoma patients has not been significantly improved for more than 20 years, which means that the current treatment of osteosarcoma has entered a bottleneck period^5^. More importantly, the survival rate of patients with advanced osteosarcoma, especially those with distant metastasis, is only about 20% even treated with chemotherapy drugs such as methotrexate, adriamycin, and cisplatin, which is far from satisfactory^6,7^. As one of the emerging cancer treatment methods, molecular targeted drugs have attracted extensive attention in recent years due to its better accuracy and less side effects. Therefore, it has become a new way to improve the prognosis of patients with osteosarcoma to identify the overexpressed marker molecules in the process of osteosarcoma progression and use them as molecular targets of targeted drugs^8,9^.

The minichromosome maintenance (MCM) complex is an important component of the prereplication complex, and is the key participant in DNA replication and extension^10,11^. The members of the MCM family include MCM2–7, as well as MCM8, MCM9, and MCM10 that have recently attracted attention^12^. MCM family proteins have similar structures, containing a conserved domain called MCM box in its central domain, which is a DNA-dependent ATPase motif composed of 200 amino acid residues. At present, the MCM complex is considered to be the helicase in the process of DNA replication, and it plays a key role in mediating the extension of the replication fork^13^. Previous studies have shown that the expression level of MCM protein and its mRNA is directly proportional to the cell proliferation activity, which also indicates that MCM protein can be used as a marker of cell proliferation to a certain extent, which has attracted the attention of tumor researchers^14^. Subsequent studies showed that MCM protein is closely related to the occurrence and development of a variety of malignant tumors^15^. Among them, a study on MCM8 showed that the expression of MCM8 was upregulated by 2–5.2-fold in human cancers including breast cancer, non-small cell lung cancer, liver cancer, glioblastoma multiforme, and myeloblastoma, indicating that overexpression of MCM8 has extensive significance in human malignant tumors^16^. Bearing all these in mind, this study aims to clarify the expression characteristics of MCM8 in osteosarcoma and its role in tumor progression.

Herein, MCM8 was identified as a key participant in the development and progression of osteosarcoma. The analysis using clinical specimens as object showed the upregulation of MCM8 in osteosarcoma and its significant association with the malignant grade and pathological stage. In vitro and in vivo investigations suggested that MCM8 silencing could be an effective strategy to suppress the development and even the metastasis of osteosarcoma. Further exploration of the downstream mechanism indicated connective tissue growth factor (CTGF) as a potential target molecule of MCM8, knockdown of which could enhance the inhibitory effects of MCM8 silencing, while weakening the promotion effects of MCM8 overexpression on osteosarcoma development.

## Materials and methods

### Immunohistochemistry (IHC) analysis

Osteosarcoma tissue and adjacent normal tissue samples were obtained from Zhengzhou University. A total of 78 cases were collected and related information of these osteosarcoma patients was collected as well. All patients were completely informed and written informed consent was provided before operation. The experimental design was approved by the institutional committees of the Animal Research Committee and Animal Ethics Committee of Zhengzhou University. For IHC, the microarrays were baked at 65 °C for 30 min, then dewaxed in xylene and hydrated in ethanol with different concentrations. Citrate buffer was used for antigen repairing at 180 °C for 5 min. After blocked with 3% H_2_O_2_, target gene antibodies (Table S1) were incubated with the microarrays at 4 °C overnight. Secondary antibody was added and incubated for 2 h at room temperature. Finally, the tissue microarrays were stained with diaminobenzidine and exanimated by microscopic. IHC scoring of specimens was classified into four categories based on the sum of the staining intensity and staining extent scores.

### Cell culture

Human osteosarcoma cell lines MNNG/HOS, Saos-2, U-2OS, and MG-63 were obtained from the Bena Technology (Hangzhou, Zhejiang, China). MNNG/HOS and MG-63 cells were maintained in 90% DMEM-H with 10% FBS additive (Gibco, Rockville, MD, USA). Saos-2 and U-2OS cells were cultured in 90% RPMI-1640 containing 10% FBS. All cells were maintained in an incubator at 37 °C with 5% CO_2_. The lentivirus with target sequences and green fluorescent protein (GFP) labeling were prepared to infect cells (at ~80% confluence). After culturing for 72 h, the cell infection efficiency was evaluated by observing GFP expression under fluorescence microscope (Olympus, Japan).

### Target RNA interferes and lentiviral vector

First, RNA interference (RNAi) specifically targeting MCM8 or CTGF were designed and synthesized by Shanghai Yibeirui Bioscienceres (Shanghai, China) (Table S2). Then, the RNAi sequences were connected to BR-V-108 or BR-V-112 lentivirus vector (Shanghai Bioscienceres, Co., Ltd), respectively. The recombined lentivirus vector plasmid together with the auxiliary plasmid (Helper 1.0 and Helper 2.0) was cotransfected to 293T cells. Then, plasmids were extracted by EndoFree maxi plasmid kit (Tiangen, Beijing, China) and qualified plasmid was packaged with virus and lentivirus quality and titer was determined.

In addition, MCM8 overexpression construct (MCM8 group, using vector as the negative control) was also designed and prepared by Shanghai Yibeirui Bioscienceres (Shanghai, China).

### RNA extraction and RT-qPCR

Cells were infected with target lentivirus or control virus and cultured for 72 h. Total RNA was extracted using TRIzol reagent (Sigma, St. Louis, MO, USA) and the quality was evaluated by Nanodrop 2000/2000C spectrophotometer (Thermo, Waltham, MA, USA) according to the manufacturer’s instructions. Two micrograms of RNA was reverse transcribed into cDNA and quantitative real-time PCR was conducted using SYBR Green Master Mixes Kit (Vazyme, Nanjing, Jiangsu, China) on the platform of VII7 Sequence Detection system (ABI, Waltham, MA, USA). GAPDH was utilized as inner control, and related primers used were showed in Table S3. The relative quantitative analysis in gene expression data were analyzed by the 2^−ΔΔCt^ method.

### Western blotting and co-IP assay

Total protein was collected after infected cells were lysed in ice-cold RIPA buffer (Millipore, Temecula, CA, USA) and the protein concentrations were detected by BCA Protein Assay Kit (HyClone-Pierce, Logan, UT, USA). Twenty micrograms of proteins were separated by 10% SDS-PAGE (Invitrogen, Carlsbad, CA, USA), then transferred onto PVDF membranes. After blocked at room temperature for 1 h with TBST solution containing 5% non‑fat milk, the membranes were incubated with primary antibodies at 4 °C overnight and continuingly incubated with the secondary antibody for 2 h at room temperature. The outcomes were visualized by enhanced chemiluminescence (ECL) (Amersham, Chicago, IL, USA).

For co-IP, 1.0 mg prepared proteins were incubated with diluted antibodies at 4 °C overnight, then the solution was incubated with 20 μL beads at 4 °C for 2 h. After centrifugation, the precipitates were cleaned with IP cracking solution, then were denatured in the IP lysate buffer and 5 × loading buffer at 100 °C boiling water for 5 min. The obtained reactants were subjected to WB assay with antibodies. Antibodies used in WB and co-IP were detailed in Table S1.

### MTT assay

Infected cells in exponential growth phase were trypsinized and seeded into a 96-well plate with 2000 cells per well and continuously incubated at 37 °C for 24, 48, 72, 96, and 120 h. Four hours before each termination, 20 μL MTT solution (5 mg/mL, Genview, El Monte, CA, USA) was added. Next, 100 μL DMSO solution was added to dissolve formazan crystal. OD490 were measured by a microplate reader (Tecan infinite, Männedorf, Zürich, Switzerland).

### Flow cytometry for apoptosis and cell cycle

Lentivirus infected cells were seeded in six-well plates in triplicate and further cultured for 5 days. Floating cells were collected, trypsinized, and washed with 4 °C ice-cold D-Hanks. After centrifugation(1000 × *g*), cells were resuspended with binding buffer, then 5 μL Annexin V-APC (eBioscience, San Diego, CA, USA) was added for staining lightless. Apoptosis analysis was measured using FACSCalibur (BD Biosciences, San Jose, CA, USA).

For cell cycle detection, prepared cells were stained by Propidium Iodide solution (Sigma, St Louis, MO, USA). Cell cycle distribution was detected by FACSCalibur and observed by micropublisher (Olympus, Tokyo, Japan).

### Wound-healing assay

Briefly, infected cells (5 × 10^4^cells/well) were seeded onto a 96-well dish. Scratches crossing the cell monolayer were made by a 96-wounding replicator (VP scientific, San Diego, CA, USA) while cell confluence reached over 90%. After rinsing gently with serum-free medium for 2–3 times, medium with 0.5% FBS was added and cultured for 20 h and photographs were taken by a fluorescence microscope at 6 and 20 h and migration rate was calculated.

### Transwell assay

Transwell assay was performed by Corning Transwell Kit (Corning, NT, USA). First, infected cells were collected, trypsinized, counted, and incubated in the upper chamber with 100 μL medium without FBS in a 24-well plate (5 × 10^4^ cells/well). Six hundred microliters of medium supplemented with 30% FBS was added in the lower chamber. After 12 or 48 h of incubation at 37 °C with 5% CO_2_, nonmetastatic cells were removed with a cotton swab. Four hundred microliters of Giemsa was added for staining and the migration ability of cells was analyzed.

### Celigo cell counting assay

Infected cells were cultured for 72 h and then the cells were seeded into 96-well plates (2000 cells/well). Cells were further cultured in MEM (10% FBS) at 37°C with 5% CO_2_ for 5 days. MEM medium was changed every 3 days. Celigo image cytometer (Nexcelom Bioscience, Lawrence, MA, USA) was applied for cell counting at days 1, 2, 3, 4, and 5 and the cell proliferation curve of 5 days was drawn.

### Colony formation assay

Infected cells in the logarithmic growth phase were seeded into six-well plates (1000 cells/well) in triplicate and further cultured for 8 days with the culture medium exchanged every 3 days. Cell clones were photographed under a fluorescence microscope. Next, all clones were fixed by 4% paraformaldehyde, stained by Giemsa, and photographed with a digital camera. Colony forming rate = (colony number/inoculated cell number) × 100%.

### PrimeView Human Gene Expression Array

Gene expression profile in infected MNNG/HOS cells was detected by PrimeView Human Gene Expression Array. Total RNA was extracted using TRIzol method. RNA concentration and values of A260/A280 were determined by Nanodrop 2000 (Thermo, Waltham, MA, USA). Gene expression array was performed with Affymetrix human GeneChip PrimeView according to the manufacturer’s instruction and the outcomes were scanned by Affymetrix Scanner 3000 (Affymetrix, Santa Clara, CA, USA). Raw data statistical significance assessment was accomplished using a Welch *t*-test with Benjamini–Hochberg FDR (|fold change| ≥ 1.3 and FDR < 0.05 as significant). Significant difference analysis and functional analysis based on ingenuity pathway analysis (IPA) (Qiagen, Hilden, Germany) was executed, and |*Z*-score | > 2 is considered meaningful.

### Human apoptosis antibody array

Briefly, proteins from infected MNNG/HOS cells were collected and the concentrations were measured by BCA Protein Assay Kit (HyClone-Pierce, Logan, UT, USA). Each array antibody membrane was blocked, then incubated with protein samples (0.5 mg/mL) overnight at 4 °C, and continuingly incubated with HRP linked Streptavidin conjugate for 1 h. ECL (Amersham, Chicago, IL, USA) was used for visualizing and the signal the spots. Gray was viewed by ImageJ and analyzed.

### Animal experiments

Our animal study was reviewed and approved by the institutional committees of the Animal Research Committee and Animal Ethics Committee of Zhengzhou University. Female 4-week-old BALB/c nude mice were purchased from Beijing Charles River Experimental Animals Co., Ltd (Beijing, China) and housed at 24 °C with a 12 h light/dark cycle controlled condition. 0.2 mL shMCM8 or shCtrl MNNG/HOS cell suspension (2 × 10^7^ cells/mL) was subcutaneously injected into 20 mice which were randomly divided into two groups (shMCM8 group and shCtrl group) for tumor formation. The growth of tumor was monitored and longest dimension (*L*) and dimension perpendicular to length (*W*) was record once a week to calculate tumor volume (*V* = π/6 × *L* × *W*^2^). In vivo fluorescence images were captured applying IVIS Spectrum Imaging System (Perkin Elmer, Waltham, MA, USA). Then, all mice were sacrificed and the tumor tissues were removed for Ki-67 immunostaining using Ki-67 antibody. Stained slides were examined with a microscopic at 100× and 200× objective lens.

### Statistical analysis

Data are expressed as the mean ± SD and *P* values are determined using Student’s *t* test with *P* < 0.05 considered statistically significant. All statistical analysis was performed using SPSS 17.0 (IBM, SPSS, Chicago, IL, USA) and GraphPad Prism 6.01 (GraphPad Software, La Jolla, CA, USA). Multiple groups were compared by one-way ANOVA. MCM8 expression difference between osteosarcoma tissues and adjacent normal tissues were analyzed with rank sum test analysis. The relation of MCM8 expression and tumor characteristics was analyzed with Mann–Whitney *U* analysis and Spearman rank correlation analysis.

## Results

### MCM8 was highly expressed in osteosarcoma tissues and cells, and linked to the pathological stage

**Fig. 1:**
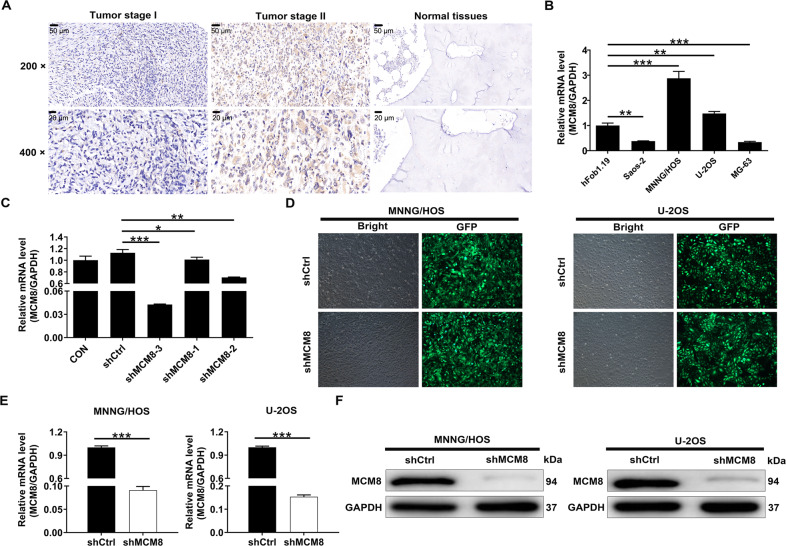
MCM8 was upregulated in osteosarcoma and MCM8 knockdown cell model was constructed. **A** The expression levels of MCM8 in osteosarcoma tumor tissues and paracarcinoma tissues were determined by immunohistochemical staining. **B** The expression levels of MCM8 in hFOB1.19 cell line and osteosarcoma cell lines were determined by qRT-PCR. **C** The knockdown efficiencies of MCM8 in MNNG/HOS cells were evaluated by qRT-PCR. **D** The transfection efficiencies of shMCM8 in MNNG/HOS and U-2OS cells were evaluated through observing the fluorescence of GFP. Magnification times: ×200. **E** The MCM8 expression in osteosarcoma cell lines after transfection was analyzed by qRT-PCR. **F** The expression of MCM8 protein in osteosarcoma cell lines after transfection was detected by western blot. Results were presented as mean ± SD. *P < 0.05, **P < 0.01, ***P < 0.001.

With the objective of determining the roles of MCM8 in osteosarcoma, we evaluated the differential expression of MCM8 between normal and osteosarcoma tissues, and between stages of disease. IHC results confirmed significantly higher expression of MCM8 in tumor tissues than in adjacent normal tissues (Fig. 1A and Table 1). The Mann–Whitney *U* analysis of the correlation between tumor characteristics and MCM8 expression in clinical samples of osteosarcoma showed that MCM8 was positively correlated to age, pathological stage, and grade malignancy (Table 2), which was further verified by the Pearson correlation analysis (Table S4). For performing experiments to study mechanism, we evaluated MCM8 levels in human osteoblast hFOB1.19 and a panel of osteosarcoma cell lines so as to find the most appropriate model (*P* < 0.001, Fig. 1B). Since the relatively higher expression was observed in the MNNG/HOS and U-2OS cells, we selected these both cell lines for application in the subsequent experiments.

**Table 1:** Expression patterns of MCM8 in osteosarcoma tissues and paracarcinoma tissues revealed in immunohistochemistry analysis.

MCM8 expression	Tumor tissue		Normal tissue		*P* value
	Cases	Percentage	Cases	Percentage	
Low	33	42.3%	21	100%	<0.001
High	45	57.7%	0	–

**Table 2:** Relationship between MCM8 expression and tumor characteristics in patients with osteosarcoma.

Features	No. of patients	MCM8 expression		*P* value
		Low	High	
All patients	76	31	45	
Age (years)				0.011
<26	38	21	17	
≥26	38	10	28	
Gender				0.555
Male	51	22	29	
Female	25	9	16	
Grade malignancy				<0.001
G2	41	25	16	
G3	35	6	29	
T Infiltrate				0.422
T1	13	4	9	
T2	63	27	36	
Lymphatic metastasis (*n*)				0.790
N0	74	30	44	
N1	2	1	1	
Stage				<0.001
1	41	25	16	
2	33	5	28	
4	2	1	1	

### MCM8 depletion reduced osteosarcoma cell proliferation, migration, and invasion but ameliorated apoptosis and cycle in vitro

Aiming to further explore the roles of MCM8 in osteosarcoma, MNNG/HOS cells were transfected with the lentivirus plasmids (shMCM8 or shCtrl). As expected, the expression of MCM8 was significantly decreased, especially in shMCM8-3 group (Fig. 1C). In addition, the fluorescence of cells, which were transfected with shMCM8-3 for 72 h, observed by microscope demonstrated a >80% efficiency of transfection (Fig. 1D). At the same time, the results of qRT-PCR and western blot indicated that after the transfection of shMCM8-3, compared with the shCtrl group, both mRNA and protein levels of MCM8 in shMCM8 group were downregulated (*P* < 0.001, Fig. 1E, F).

**Fig. 2:**
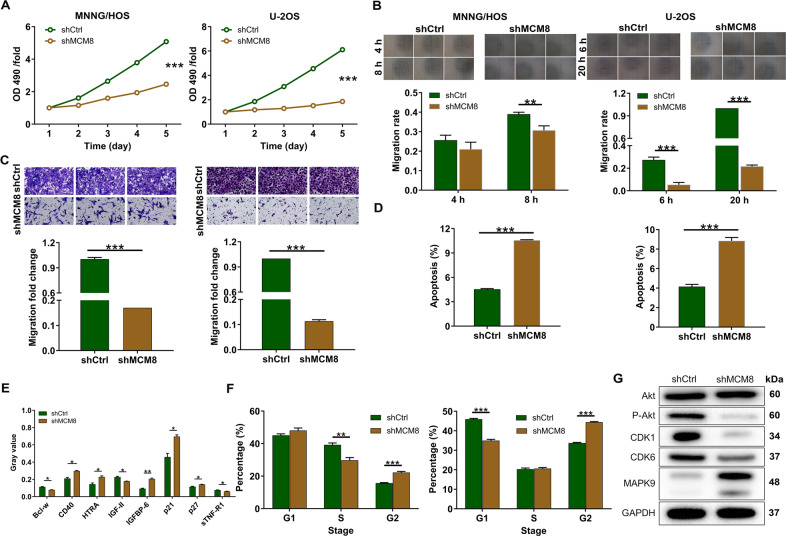
MCM8 knockdown inhibited osteosarcoma development in vitro. **A** The cell proliferation rate was evaluated in osteosarcoma cell lines after transfection by MTT assay. **B** The migration rate of cells was detected in osteosarcoma cell lines after transfection by wound-healing assay. **C** The migration rate of cells was detected in osteosarcoma cell lines after transfection by transwell assay. Magnification times: ×200. **D** The effects of MCM8 knockdown on cell apoptosis were examined by flow cytometry. **E** The expression levels of apoptosis-related proteins were analyzed in osteosarcoma cells with shMCM8. **F** The effects of MCM8 knockdown on cell cycle were determined by flow cytometry. Results were presented as mean ± SD. **G** The expression of Akt, P-Akt, CDK1, MAPK9, and CDK6 was detected by western blot in MNNG/HOS cells of shCtrl and shMCM8 groups. *P < 0.05, **P < 0.01, ***P < 0.001.

We next examined whether MCM8 depletion altered the proliferation of osteosarcoma cell lines. It was evident that MCM8 depletion in MNNG/HOS and U-2OS osteosarcoma cell lines significantly decreased cell proliferation (*P* < 0.001, Fig. 2A). The wound-healing assay and transwell assay demonstrated that the migration and invasion of MNNG/HOS and U-2OS cells were significantly slowed upon transfection of shMCM8-3 (*P* < 0.001, Fig. 2B, C). The results from flow cytometry showed that MCM8 depletion significantly accelerated the apoptosis of MNNG/HOS and U-2OS cells (*P* < 0.001, Fig. 2D), which was caused by the regulation of apoptosis-related proteins. In detail, the results of a human apoptosis antibody array showed that the expression patterns of CD40, HTRA, IGFBP-6, p21, and p27 were augmented, while those of Bcl-w, IGF-II, and sTNF-R1 were decreased after silencing MCM8 (Fig. 2E, Supplementary Fig. 1A, B). Besides, the cell cycle analysis showed that MCM8 depletion increased the percentage of cells in G2 phase in both MNNG/HOS and U-2OS cells (*P* < 0.001, Fig. 2F). Some cancer-related proteins were consistently changed in MNNG/HOS cells after knocking down MCM8, including the downregulation of P-Akt, CDK1, and CDK6, as well as the upregulation ofMAPK9 (Fig. 2G). As per the results presented so far, we indicated that MCM8 downregulation repressed proliferation, migration, and invasion but promoted apoptosis and cycle of osteosarcoma cells.

### MCM8 depletion curbed osteosarcoma tumorigenesis in vivo

**Fig. 3:**
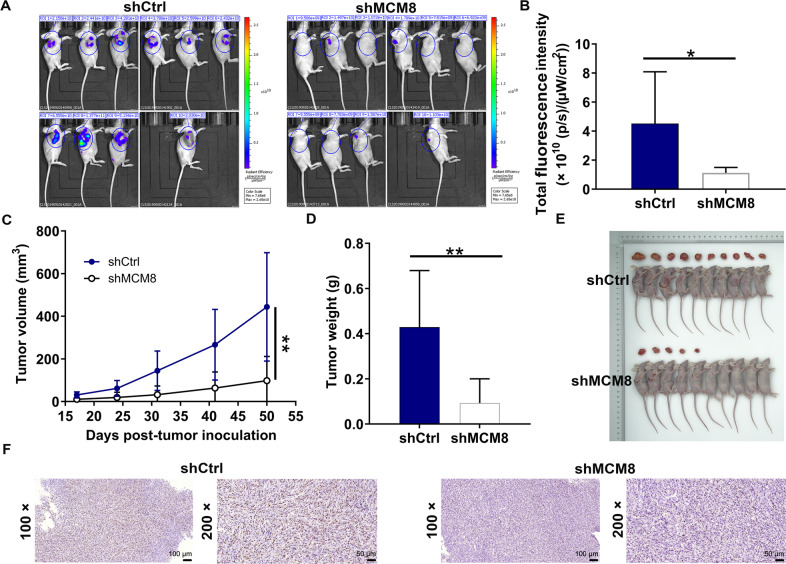
MCM8 knockdown suppressed osteosarcoma growth in vivo. **A** A nude mice model of MCM8 knockdown through injecting MNNG/HOS cells was constructed. **B** The fluorescence intensity was obtained through injection of D-Luciferase before sacrificing the mice. **C** The volume of tumors was tested from feeding to sacrifice. **D** The weight of tumors was measured after sacrificing mice. **E** The photograph of tumors was taken after removing tumors. **F** The value of Ki-67 was detected by IHC in tumor sections. Results were presented as mean ± SD. *P < 0.05, **P < 0.01.

After establishing a possible involvement of MCM8 in osteosarcoma in vitro, we turned to in vivo models for further verification. We injected MNNG/HOS cells with MCM8 depletion subcutaneously in mice, which was observed by in vivo imaging (Fig. 3A). Such decrease in the fluorescence was considered to be impaired tumor growth in shMCM8 group (*P* < 0.05, Fig. 3B). We indeed found significant reduction in tumor volume and weight (*P* < 0.01, Fig. 3C–E). Finally, the expression patterns of factors related to proliferation in tumor tissues were determined. The results showed that MCM8 depletion led to diminished expression of Ki-67 (Fig. 3F). As presented above, these data suggested that MNNG/HOS cells with MCM8 depletion curbed osteosarcoma tumorigenesis as xenografts in nude mice.

### MCM8 depletion inhibited osteosarcoma development through CTGF

**Fig. 4:**
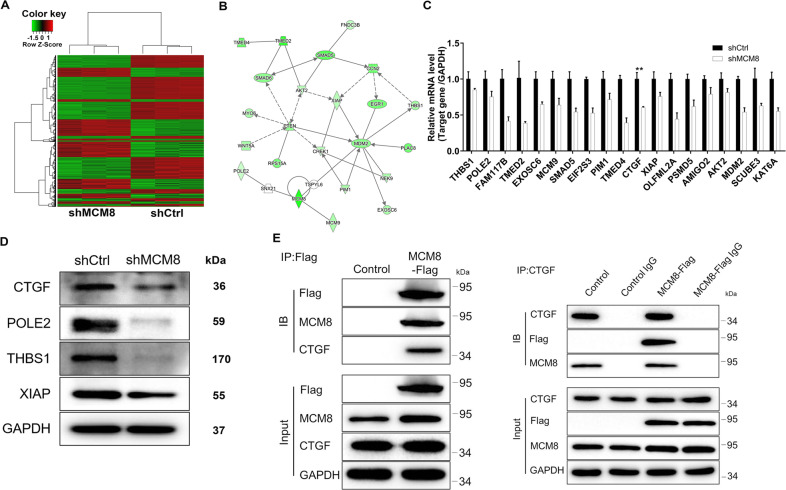
Exploration of underlying mechanism by GeneChip and IPA analysis. **A** The heatmap of DEGs identified by RNA-sequencing of cells treated with shCtrl (*n* = 3) or shMCM8 (*n* = 3). **B** Interaction network diagram between DEGs was analyzed by IPA. The expression of several most significantly differentially expressed genes identified by qRT-PCR (**C**) and western blot (**D**) in MNNG/HOS cells with shMCM8. **E** Co-IP assay was used to verify whether there was protein interaction between MCM8 and CTGF. Control: MNNG/HOS cells transfected with negative control lentivirus. MCM8-Flag: MNNG/HOS cells transfected with Flag tagged MCM8 overexpression lentivirus. The data are expressed as mean ± SD (*n* ≥ 3).

We were interested in finding downstream mechanism that was functionally involved in MCM8’s actions towards promoting osteosarcoma. In this regard, we turned to PrimeView Human Gene Expression Array to make clear the differentially expressed genes (DEGs) in shMCM8 and shCtrl MNNG/HOS cells. It followed that there were 629 upregulated DEGs and the other 867 downregulated DEGs in shMCM8 group based on the threshold of absolute fold change ≥ 1.3 and FDR < 0.05 (Fig. 4A, Supplementary Fig. 1C). Subsequently, all the DEGs were enriched by the interaction network analysis based on IPA disease and function, indicating the downregulation of AKT2, CTGF, CHEK1, EGR1, EXOSC6, FNDC3B, MCM9, MDM2, MYO6, NEK9, PIM1, PLAC8, POLE2, PTEN, RPS15A, SMAD5, SMAD6, THBS1, TMED2, TMED4, WNT5A, and XIAP in MCM8 depletion treatment (Fig. 4B). Among them, 19 DEGs were selected for qPCR detection in shMCM8 and shCtrl MNNG/HOS cells, 4 of which were used for the western blot analysis. The results indicated that CTGF, POLE2, THBS1, and XIAP mRNA levels (Fig. 4C) and protein levels (Fig. 4D) both exhibited a significant downward trend. Finally, in the co-IP assay, the protein obtained by coprecipitation with Flag antibody was detected by CTGF antibody, which demonstrated that the expression of CTGF protein in MCM8-Flag group was significantly increased (Fig. 4E), indicating that there was an interaction between MCM8 and CTGF. Herein, it was speculated that CTGF was the downstream target of MCM8 involved in the regulation of osteosarcoma.

### CTGF was involved in the MCM8-induced regulation of osteosarcoma

**Fig. 5:**
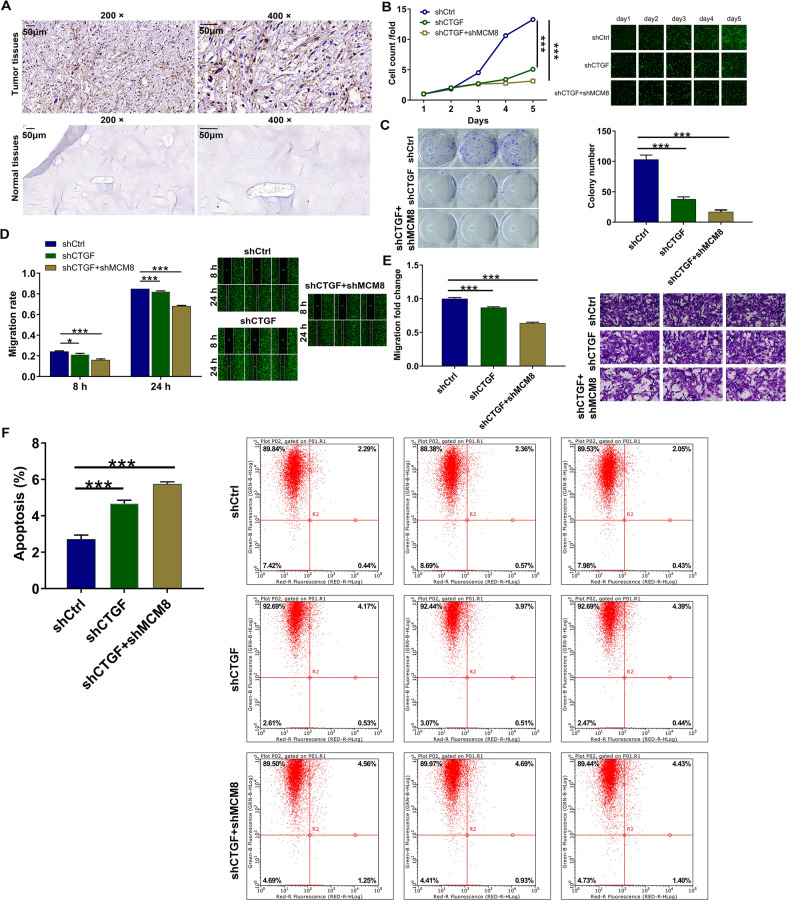
Knockdown of CTGF strengthened the suppression of osteosarcoma caused by MCM8 knockdown. **A** The expression of CTGF in tissues of normal and tumor was detected by IHC analysis. **B** Celigo cell counting assay was employed to show the effects of CTGF knockdown on MNNG/HOS cell proliferation in shCTGF and shMCM8 + shCTGF groups. **C** Colony formation assay was used to evaluate the ability of MNNG/HOS cells to form colonies in shCTGF and shMCM8 + shCTGF groups. **D** The migration rate of cells was detected in shCTGF and shMCM8 + shCTGF groups by wound-healing assay. **E** The migration rate of cells was detected in shCTGF and shMCM8 + shCTGF groups by transwell assay. Magnification times: ×200. **F** The cell apoptosis was examined by flow cytometry in shCTGF and shMCM8 + shCTGF groups. Results were presented as mean ± SD. *P < 0.05, ***P < 0.001.

**Fig. 6:**
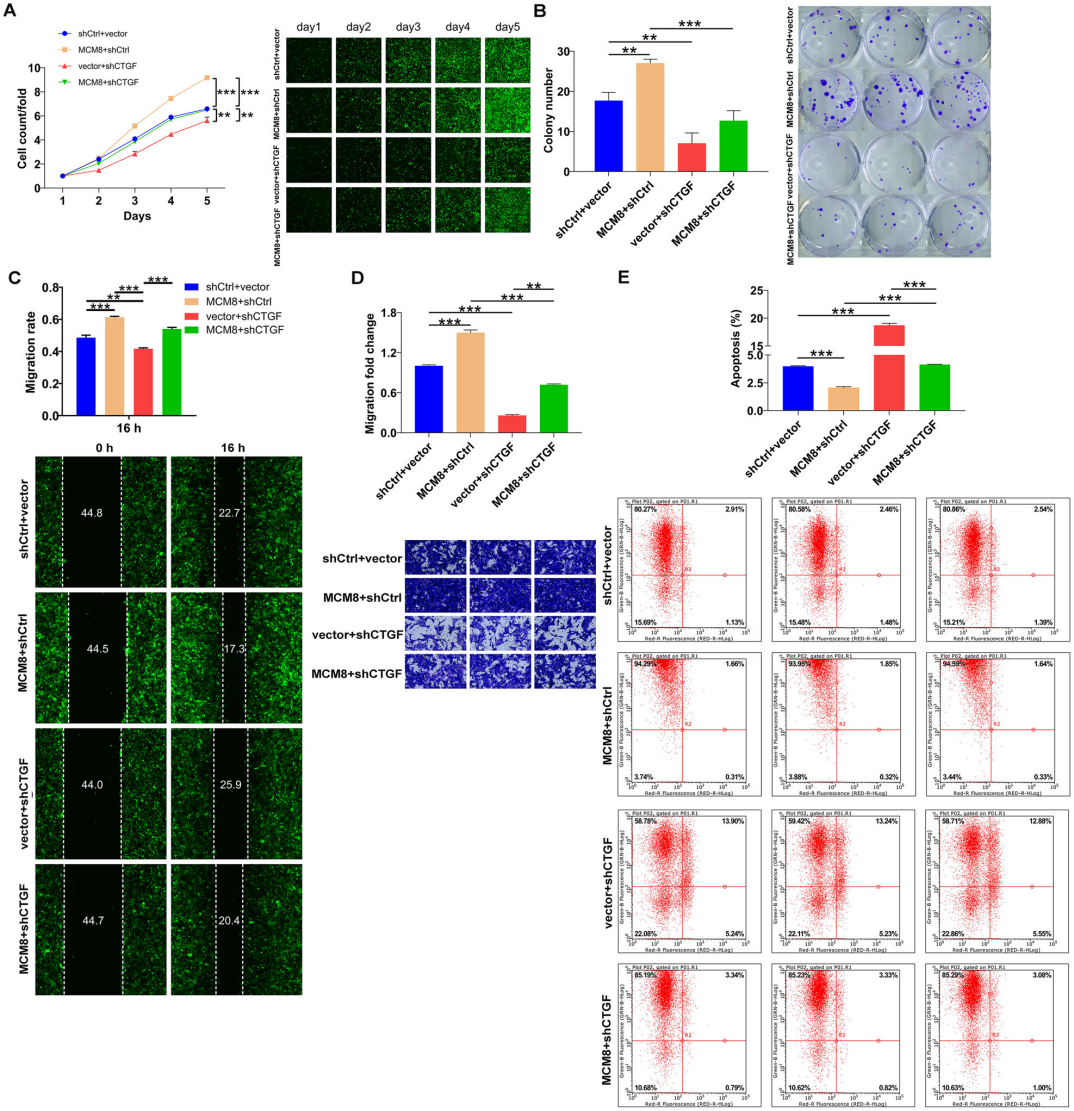
Knockdown of CTGF alleviated the promotion of osteosarcoma caused by MCM8 overexpression. **A** Celigo cell counting assay was employed to show the effects of mere MCM8 overexpression, mere CTGF knockdown and concurrent MCM8 overexpression and CTGF knockdown on cell proliferation. **B** Colony formation assay was used to evaluate the effects of mere MCM8 overexpression, mere CTGF knockdown and concurrent MCM8 overexpression and CTGF knockdown on colony formation. **C** The effects of mere MCM8 overexpression, mere CTGF knockdown and concurrent MCM8 overexpression and CTGF knockdown on cell migration were evaluated by wound-healing assay. **D** The effects of mere MCM8 overexpression, mere CTGF knockdown and concurrent MCM8 overexpression and CTGF knockdown on cell migration were evaluated by transwell assay. Magnification times: ×200. **E** The effects of mere MCM8 overexpression, mere CTGF knockdown and concurrent MCM8 overexpression and CTGF knockdown on cell apoptosis were evaluated by flow cytometry. Results were presented as mean ± SD. *P < 0.05, ***P < 0.001.

Initially, IHC results illustrated the upregulated CTGF expression in osteosarcoma tissues (Fig. 5A). Next, in an attempt to identify the synergistic function of CTGF and MCM8 in the development of osteosarcoma, MNNG/HOS cell models with mere CTGF depletion and concurrent MCM8 depletion and CTGF depletion were prepared, verified (Supplementary Fig. 2A–D), and used in the following functional experiments. It needed to be mentioned that cell phenotypes including proliferation, migration, and coupled with colony formation were impaired, especially in shCTGF + shMCM8 group (Fig. 5B–E). In addition to the alteration of cell proliferation and migration, we also tested cell apoptosis ability. Not surprisingly, the apoptosis level increased in both shCTGF and shCTGF + shMCM8 groups (*P* < 0.001, Fig. 5F). Furthermore, shCTGF was also used for transfecting MCM8 overexpression MNNG/HOS cell model to construct cell model with concurrent MCM8 overexpression and CTGF knockdown. The following detection of cell phenotypes indicated not only that MCM8 overexpression promotes cell proliferation (Fig. 6A), colony formation (Fig. 6B), and cell migration (Fig. 6C, D) as well as inhibits cell apoptosis (Fig. 6E) but also that CTGF knockdown could partially reverse the MCM8-induced regulatory effects on osteosarcoma cells (Fig. 6). Based on the results that were presented, it was quite apparent that CTGF might be a target of MCM8 in the regulation of osteosarcoma.

## Discussion

Although MCM family members were initially recognized as key factors in DNA replication and extension, their biological functions in the development and progression of human diseases including cancer have attracted considerable attention in recent years^14,15^. Considering the significance of MCM proteins as representative of cell proliferation, it was identified as a potential diagnostic biomarker of breast cancer, together with the more famous Ki-67 and PCNA^17^. Previous studies showed that phosphorylation induced by various kinases is the key to regulate the activity and function of MCM proteins. These phosphorylation events are not only related to DNA replication but also to cell cycle progression and checkpoint reaction. Therefore, abnormal phosphorylation of MCM is related to the occurrence and development of cancer^18^. For instance, phosphorylation of MCM3 by PLK1 was proved to participate in the proliferation and apoptosis of renal cell carcinoma cells^19^. On the other hand, MCM5 was also manifested as a regulator in renal cell carcinoma development which was related with poor prognosis of patients^20^. MCM7, whose amplification was known to be responsible for prostate cancer development^21^, was recently found to promote hepatocellular carcinoma development through a CyclinD1-related path^22^. Liu et al. demonstrated that MCM6 is capable of promoting the expression levels of a series of cell cycle markers including CDK2, CDK4, CyclinA, CyclinB1, CyclinD1, and CyclinE and delaying cell cycle progression in S/G2 phase. Notably, they also found upregulation of MCM8 in hepatocellular carcinoma^23^. In view of the important role of MCM8-9 complex in DNA synthesis, the latest research showed that inhibiting the formation of this complex can increase the sensitivity of tumor cells to DNA damage drugs such as cisplatin and olaparib^24^. However, research concerning the role of MCM8 itself in the development and progression of human cancers is still rarely reported, which is only observed in gastric cancer ^25^.

In this study, we found that osteosarcoma tissues possessed generally higher MCM8 expression than normal bone tissues. High MCM8 expression was significantly correlated with more advanced tumor grade and pathological stage. In consistent with the role of MCM proteins as indicator of cell proliferation, we also found that knockdown of MCM8 could impede osteosarcoma cell proliferation, together with the promotion of cell apoptosis, arrest of cell cycle in G2 phase, and suppression of cell migration. Further mechanistic study revealed that MCM8 may regulate a variety of apoptosis-related proteins and Akt pathway to influence osteosarcoma development. We also use xenograft mice model to demonstrate the knockdown of MCM8 as a strategy to inhibit osteosarcoma development in vivo.

Furthermore, a GeneChip microarray analysis, followed by bioinformatics analysis, revealed CTGF as a potential downstream molecule of MCM8 with direct protein–protein interaction. Subsequent verification showed that CTGF exhibited a feature of coexpression with MCM8 in osteosarcoma tissues or cells. Knockdown of CTGF could deepen the regulatory effects of MCM8 silencing on osteosarcoma development. More importantly, upon CTGF knockdown, the MCM8-induced promotion of osteosarcoma development was significantly alleviated.

CTGF is a secreted peptide rich in cysteine, composed of 349 amino acids, and plays an important role in the process of cell hypertrophy and proliferation and the synthesis of extracellular matrix components. CTGF exists in lung, brain, liver, and other tissues, which regulates the physiological functions of cells by interacting with different ligands and integrins^26,27^. For example, the combination of CTGF and integrin α6β1 can effectively promote the adhesion, migration, and survival of epithelial cells and microvascular endothelial cells^28^; the combination of CTGF and IGF1 could regulate the proliferation and development of chondrocytes^29^; the interaction of CTGF and LRP can regulate cell adhesion and Wnt signal pathways^30^, etc. Although the initial study found that CTGF has chemotaxis and can promote the mitosis of fibroblasts, a large number of studies in recent years have shown that it plays an important role in the fibrosis of many tissues and organs throughout the body^31–33^. More importantly, recent research showed that CTGF also plays a critical role in the development of human cancers^34^. For example, it was illustrated that CTGF mediated the regulation of epithelial–mesenchymal transition and angiogenesis of colorectal cancer by miR-218^35^, and the promotion of pancreatic cancer by PD-1^36^. Recently, CTGF was also found to play an important role in the growth of bone metastases from prostate and breast cancer through forming CTGF–Runx2–RANKL axis^37^. Moreover, the role of CTGF in osteosarcoma to be used was demonstrated by Wang et al., as a promotor of angiogenesis^38^, and by Hou et al., for promoting tumor metastasis through regulating VCAM-1^39^. Herein, MCM8/CTGF turned out to be a new path that CTGF executes its regulatory effects in osteosarcoma.

In conclusion, the results of our study demonstrated that MCM8 is significantly associated with the promotion of cell proliferation and cell migration, and the inhibition of cell apoptosis in osteosarcoma, thus predicting more advanced tumor grade and pathological stage. Moreover, CTGF, whose effects of osteosarcoma have been reported to some extent, was revealed as a potential downstream target of MCM8. Dual target of MCM8 and CTGF exhibited stronger inhibitory effects on osteosarcoma development than mere MCM8 knockdown. Therefore, MCM8 may be considered as a novel therapeutic target of osteosarcoma.

## Supplementary information

Supplementary figure legends

Figure S1

Figure S2

Table S1

Table S2

Table S3

Table S4
