# Supplementary material for: Knockdown of MCM8 functions as a strategy to inhibit the development and progression of osteosarcoma through regulating CTGF
Source: Cell Death Dis. 2021 Apr 7;12(4):376. doi: 10.1038/s41419-021-03621-y (PMC8027380; doi:10.1038/s41419-021-03621-y)
Supplement: Supplementary file 1 — Supplementary figure legends [file 41419_2021_3621_MOESM1_ESM.docx]

**Figure S1.** (A) The expression of apoptosis-related proteins in MNNG/HOS cells transfected with shMCM8 was measured by ECL with Human Apoptosis Antibody Array. The results circled in red indicated that the protein expression was up-regulated and *P* < 0.05. (B) Protein expression was presented in grayscale and visualized by R studio. Results were presented as mean ± SD. (C) The volcano plot of gene expression profiling in MNNG/HOS cells with or without MCM8 knockdown. Red dots represent the upregulated DEGs, green dots represent the downregulated DEGs.

**Figure S2.** (A) The knockdown efficiencies of CTGF in MNNG/HOS cells were evaluated by qRT-PCR. (B) The transfection efficiencies of shCTGF and shMCM8+shCTGF in MNNG/HOS cells were evaluated through observing the fluorescence of GFP. Magniﬁcation times: 200×. (C, D) The mRNA and protein levels of MCM8 and CTGF in shCTGF, shMCM8+shCTGF and shCtrl groups of MNNG/HOS cells were detected by qRT-PCR (C) and WB (D).
