# Supplementary figures and images for: Knockdown of MCM8 functions as a strategy to inhibit the development and progression of osteosarcoma through regulating CTGF
Source: Cell Death Dis. 2021 Apr 7;12(4):376. doi: 10.1038/s41419-021-03621-y (PMC8027380; doi:10.1038/s41419-021-03621-y)

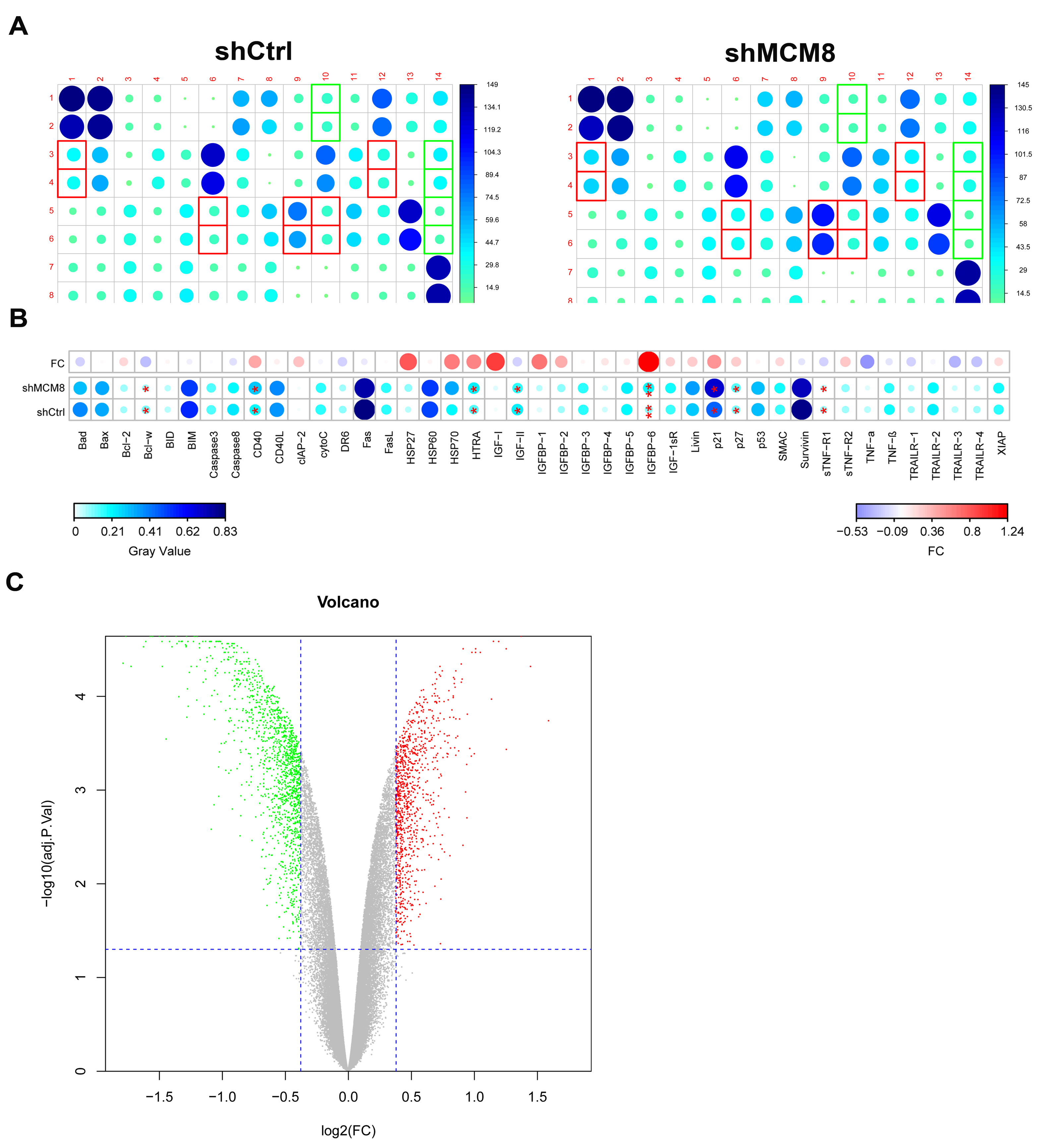

Supplement: Supplementary file 2 — Figure S1 [file 41419_2021_3621_MOESM2_ESM.tif]

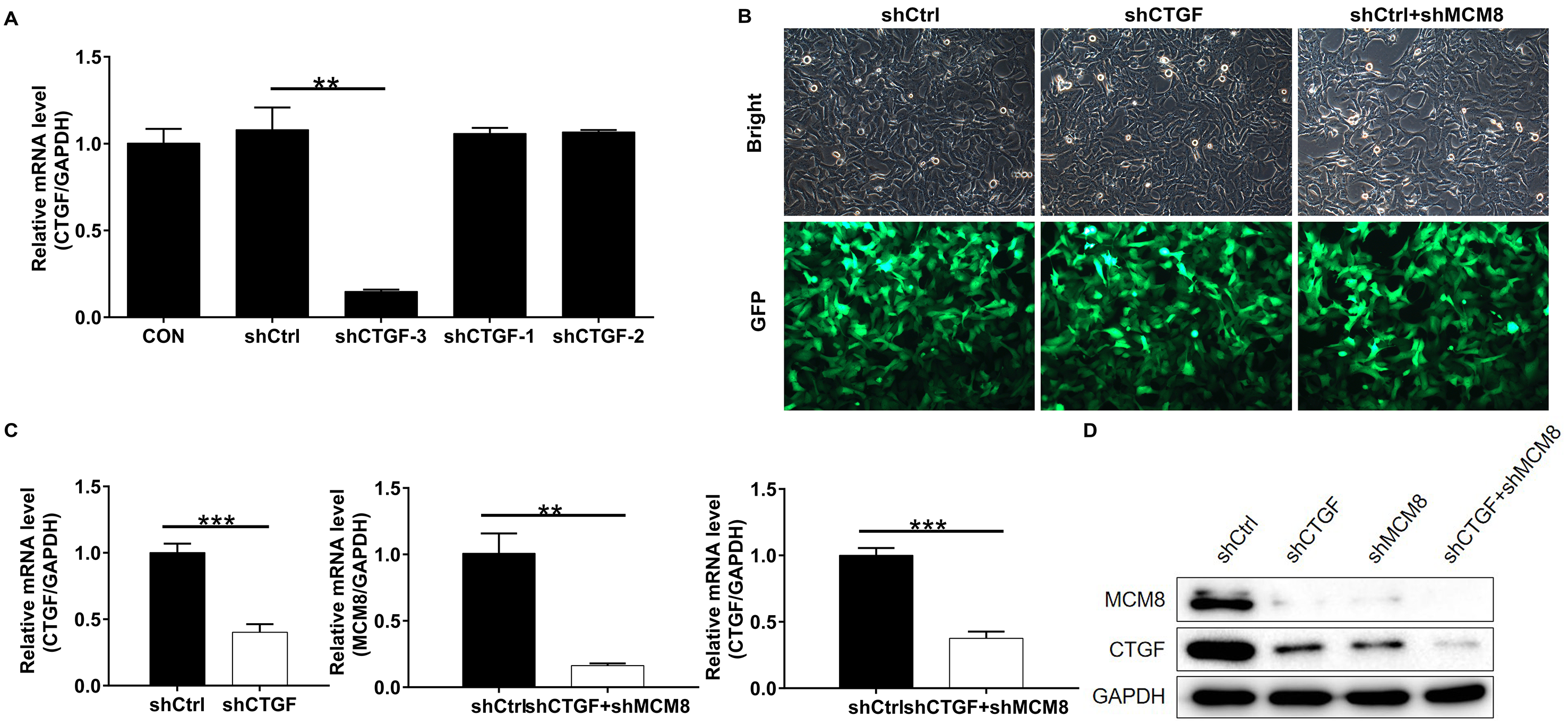

Supplement: Supplementary file 3 — Figure S2 [file 41419_2021_3621_MOESM3_ESM.tif]
