# Supplementary material for: Knockdown of MCM8 functions as a strategy to inhibit the development and progression of osteosarcoma through regulating CTGF
Source: Cell Death Dis. 2021 Apr 7;12(4):376. doi: 10.1038/s41419-021-03621-y (PMC8027380; doi:10.1038/s41419-021-03621-y)
Supplement: Supplementary file 4 — Table S1 [file 41419_2021_3621_MOESM4_ESM.docx]

Table S1 Antibodies used in western blotting and IHC

| Primary antibodies | Dilution in WB | Source species | Company | Catalog No. |
| --- | --- | --- | --- | --- |
| MCM8 | 1:1000 | Rabbit | Invitrogen | PA5-41325 |
| GAPDH | 1:3000 | Rabbit | Bioworld | AP0063 |
| Akt | 1:1000 | Rabbit | CST | 4685 |
| P-Akt | 1:500 | Rabbit | R&D | AF887-sp |
| CDK1 | 1:3000 | Rabbit | bioss | bs-0542R |
| CDK6 | 1:1000 | Rabbit | abcam | ab151247 |
| MAPK9 | 1:3000 | Rabbit | abcam | ab76125 |
| CTGF | 1:1000 | Rabbit | abcam | ab6992 |
| POLE2 | 1:1000 | Rabbit | abcam | ab180214 |
| THBS1 | 1:1000 | Rabbit | CST | 37879 |
| XIAP | 1:1000 | Mouse | abcam | ab28151 |
| DYKDDDDK Tag | 1:50/1:1000 | Rabbit | CST | 14793 |
| Primary antibodies | Dilution in IHC | Source species | Company | Catalog No. |
| MCM8 | 1:100 | Rabbit | Invitrogen | PA5-41325 |
| CTGF | 1:200 | Rabbit | abcam | ab6992 |
| Ki67 | 1:200 | Rabbit | abcam | ab16667 |
|  |  |  |  |  |
|  |  |  |  |  |
| Secondary antibody | Dilution |  | Company | Catalog No. |
| HRP Goat Anti-Rabbit IgG (WB) | 1:3000 |  | Beyotime | A0208 |
| HRP Goat Anti-Mouse IgG (WB) | 1:3000 |  | Beyotime | A0216 |
| HRP Goat Anti-Rabbit IgG (IHC) | 1:400 |  | Abcam | Ab6721 |
