# Supplementary material for: Knockdown of MCM8 functions as a strategy to inhibit the development and progression of osteosarcoma through regulating CTGF
Source: Cell Death Dis. 2021 Apr 7;12(4):376. doi: 10.1038/s41419-021-03621-y (PMC8027380; doi:10.1038/s41419-021-03621-y)
Supplement: Supplementary file 5 — Table S2 [file 41419_2021_3621_MOESM5_ESM.docx]

Table S2 The target sequences and shRNA sequences

| Gene | No. | Target sequence (5'-3') | shRNA sequences (5'-3') |
| --- | --- | --- | --- |
| MCM8 | Pbr-11106-a | TGGCAATACATCAGGTGTTAA | CcggTGGCAATACATCAGGTGTTAActcgagTTAACACCTGATGTATTGCCATTTTTg |
| MCM8 | Pbr-11106-b | TGGCAATACATCAGGTGTTAA | aattcaaaaaTGGCAATACATCAGGTGTTAActcgagTTAACACCTGATGTATTGCCA |
| MCM8 | Pbr-11107-a | CTGGAATTGTCAAAGTCTCAA | CcggCTGGAATTGTCAAAGTCTCAActcgagTTGAGACTTTGACAATTCCAGTTTTTg |
| MCM8 | Pbr-11107-b | CTGGAATTGTCAAAGTCTCAA | aattcaaaaaCTGGAATTGTCAAAGTCTCAActcgagTTGAGACTTTGACAATTCCAG |
| MCM8 | Pbr-11108-a | AGGCAGCTGGAATCTTTGATT | CcggAGGCAGCTGGAATCTTTGATTctcgagAATCAAAGATTCCAGCTGCCTTTTTTg |
| MCM8 | Pbr-11108-b | AGGCAGCTGGAATCTTTGATT | aattcaaaaaAGGCAGCTGGAATCTTTGATTctcgagAATCAAAGATTCCAGCTGCCT |
| CTGF | Pbr12886-a | CGAAGCTGACCTGGAAGAGAA | CCGGCGAAGCTGACCTGGAAGAGAACTCGAGTTCTCTTCCAGGTCAGCTTCGTTTTTG |
| CTGF | Pbr12886-b | CGAAGCTGACCTGGAAGAGAA | AATTCAAAAACGAAGCTGACCTGGAAGAGAACTCGAGTTCTCTTCCAGGTCAGCTTCG |
| CTGF | Pbr12887-a | CTACAGGAAGATGTACGGAGA | CCGGCTACAGGAAGATGTACGGAGACTCGAGTCTCCGTACATCTTCCTGTAGTTTTTG |
| CTGF | Pbr12887-b | CTACAGGAAGATGTACGGAGA | AATTCAAAAACTACAGGAAGATGTACGGAGACTCGAGTCTCCGTACATCTTCCTGTAG |
| CTGF | Pbr12888-a | CGCGGCTTACCGACTGGAAGA | CCGGCGCGGCTTACCGACTGGAAGACTCGAGTCTTCCAGTCGGTAAGCCGCGTTTTTG |
| CTGF | Pbr12888-b | CGCGGCTTACCGACTGGAAGA | AATTCAAAAACGCGGCTTACCGACTGGAAGACTCGAGTCTTCCAGTCGGTAAGCCGCG |
|  |  |  |  |
