# Supplementary material for: Knockdown of MCM8 functions as a strategy to inhibit the development and progression of osteosarcoma through regulating CTGF
Source: Cell Death Dis. 2021 Apr 7;12(4):376. doi: 10.1038/s41419-021-03621-y (PMC8027380; doi:10.1038/s41419-021-03621-y)
Supplement: Supplementary file 6 — Table S3 [file 41419_2021_3621_MOESM6_ESM.docx]

Table S3 Primers used in qPCR

| Gene | Forward primer sequence (5’-3’) | Reverse primer sequence (5’-3’) |
| --- | --- | --- |
| GAPDH | TGACTTCAACAGCGACACCCA | CACCCTGTTGCTGTAGCCAAA |
| MCM8 | ATGGCTTTTCTTTGTGCTGC | CCAGTCCATCGTAACTGTGAGA |
| THBS1 | TGTTCTCTACTGGCTTTATGTCA | GGCCTGAGCAACTCAGTCTT |
| POLE2 | TGAGAAGCAACCCTTGTCATC | TCATCAACAGACTGACTGCATTC |
| FAM117B | CAGAGCCGAAGCTCGCCGGAGAAGA | CAGGGAGGAAGTGCGTCGGATAATACTGG |
| TMED2 | TGGTGACGCTTGCTGAACTG | AGATGAGGCCCATCTTGGTG |
| EXOSC6 | GAAGCCTGAGCAACTACGGA | AATTCTCGACGCAAACTGGA |
| MCM9 | GGTTTGCCTGTCTGTCCTGA | GTAATCCCGCTCAAACTCCA |
| SMAD5 | GCTTCTGGCTCAATCTGTCAAC | GATATTCTGCTCCCCAACCCT |
| EIF2S3 | GCCCCGGCTTATTGTTATT | ACCACCAGCTACACCTCCCTTA |
| PIM1 | TTTTCTTCAGGCAGAGGGTCT | GGAGGTGGATCTCAGCAGTTT |
| TMED4 | CCAGGGGCTCTACTTCCACA | ACGATAGTTGCCGATGACCA |
| CTGF | GGTGTGGCTTTAGGAGCAGT | TGATGGCTGGAGAATGCACA |
| XIAP | CTATGCTCACCTAACCCCAAGA | GTGTTCTGACCAGGCACGAT |
| OLFML2A | CGGGAGAATGAGGTGGTGAA | CCAGCATGATGGCAGAGTGA |
| PSMD5 | AAATTCTGATGCTGTTACTGA | AAAGATTCTGGTGACACGGA |
| AMIGO2 | CGGATTTCATCTGGGTGGGT | CCTCAAAACGAGGGCTTTCTAT |
| AKT2 | CCTCATGCTGGACAAAGATGG | GGGGTCCCACAGAAGGTTTT |
| MDM2 | GGTGAGGAGCAGGCAAATGT | CGAAGCTGGAATCTGTGAGGT |
| SCUBE3 | CAGCACCAACTCCAACCACT | AACGGCATTTGGCATCCT |
| KAT6A | GCCAATGCCAAGATTAGAACC | CGAAGACAAACGACGGAAGTAT |
|  |  |  |
