# Supplementary material for: Knockdown of MCM8 functions as a strategy to inhibit the development and progression of osteosarcoma through regulating CTGF
Source: Cell Death Dis. 2021 Apr 7;12(4):376. doi: 10.1038/s41419-021-03621-y (PMC8027380; doi:10.1038/s41419-021-03621-y)
Supplement: Supplementary file 7 — Table S4 [file 41419_2021_3621_MOESM7_ESM.docx]

Table S4. Relationship between MCM8 expression and tumor characteristics in patients with osteosarcoma.

|  |  | MCM8 |
| --- | --- | --- |
| Grade malignancy | Pearson correlation | 0.445 |
|  | Signification (double-tailed) | <0.001 |
|  | N | 76 |
| Stage | Pearson correlation | 0.424 |
|  | Signification (double-tailed) | <0.001 |
|  | N | 76 |
